# Estimated Clinical Trial Capacity of Sites Participating in the COVID-19 Convalescent Plasma Expanded Access Program

**DOI:** 10.1001/jamanetworkopen.2022.37540

**Published:** 2022-10-19

**Authors:** M. Sage Gustafson, Aman Patel, Chuan Hong, Miles Meline, Daniella Peña, Charis Tang, Holly Fernandez Lynch

**Affiliations:** 1 Division of Medical Ethics, NYU Langone Health, New York, New York; 2 University of Southern California, Los Angeles, California; 3 Department of Population Health, NYU Langone Health, New York, New York; 4 University of Pennsylvania, Philadelphia, Pennsylvania; 5 Cornell University, Ithaca, New York; 6 Rice University, Cupertino, California; 7 Department of Medical Ethics and Health Policy Perelman School of Medicine, University of Pennsylvania, Philadelphia, Pennsylvania

## Abstract

This cross-sectional study estimates the trial capacity of sites participating in the COVID-19 convalescent plasma expanded access program.

## Introduction

Expanded access (EA) allows patients to receive unapproved medicines outside trials only if doing so would not interfere with drug development. However, interference may not be obvious: is EA needed because a site lacks trial capacity, or is EA inhibiting trial initiation? These questions were highlighted in the context of the largest-ever EA program (EAP), implemented for COVID-19 convalescent plasma (CCP). The program was initially planned for 5000 patients but ultimately enrolled 105 717 patients.^1^ We sought to estimate the trial capacity of participating CCP EAP sites, hypothesizing that many sites could have supported CCP research to better guide clinical care.

## Methods

For this cross-sectional study, we used ClinicalTrials.gov to determine the number and type of trials initiated at EAP sites during the 2 years before the pandemic (March 1, 2018, to February 29, 2020), as well as COVID-19 trials initiated at these sites before CCP was granted emergency use authorization (March 1 to August 23, 2020). We designed an algorithm using Google to assess equivalence between ClinicalTrials.gov locations and EAP site names,^1^ manually validating a subset for accuracy (*f*_β_ = 0.91). Data, code, and additional methods meeting the STROBE reporting guideline are posted on Open Science Framework.^2^ Because this project did not involve human participants, institutional review board approval was not required.

## Results

Of 2239 EAP sites, we identified 1246 (55.7%) that started at least 1 trial between March 1, 2018, and February 29, 2020 (Table). During that period, 1018 sites (45.5%) initiated at least 1 interventional randomized drug or biologic clinical trial, and 408 sites (18.2%) initiated at least 1 interventional randomized infectious disease clinical trial. Almost one-third of EAP sites (701 [31.3%]) initiated at least 1 COVID-19 trial before emergency use authorization. Approximately half of those sites (378 [16.9%]) initiated at least 1 interventional randomized clinical trial investigating COVID-19 drugs or biologics, with most of these sites (308 [13.8%]) initiating at least 1 such phase 3 trial. A small percentage, but substantial number, of sites (78 [3.5%]) initiated at least 1 interventional randomized clinical trial of CCP in addition to EAP participation (Figure). Thirty-six of the top 100 NIH-funded sites in fiscal year 2020 participated in the EAP.^2^

**Table.** Relevant Trial Types Initiated at CCP EAP Sites Before and During the COVID-19 Pandemic

Trial types	CCP EAP sites, No. (%) (N = 2239)
	
General (non–COVID-19) trials (March 1, 2018, to February 29, 2020)	
Sites with ≥1 trial	1246 (55.7)
Sites with ≥1 randomized interventional drug or biologic clinical trial	1018 (45.5)
Sites with ≥1 randomized interventional, phase 3 drug or biologic clinical trial	930 (41.5)
Sites with ≥1 randomized interventional infectious disease clinical trial	408 (18.2)
Top-funded NIH sites with ≥100 interventional trials, No./total No. (%)^a^	27/36 (75.0)^b^
Top-funded NIH sites with ≥1 randomized interventional infectious disease clinical trial, No./total No. (%)^a^	33/36 (91.7)^b^
COVID-19 trials (March 1 to August 23, 2020)	
Sites with ≥1 trial	701 (31.3)
Sites with ≥1 randomized interventional drug or biologic clinical trial	378 (16.9)
Sites with ≥1 randomized interventional, phase 3 drug or biologic clinical trial	308 (13.8)
Sites with ≥1 randomized interventional CCP clinical trial	78 (3.5)
Top-funded NIH sites with ≥1 randomized interventional drug or biologic clinical trial, No./total No. (%)^a^	35/36 (97.2)^b^
Top-funded NIH sites with ≥1 randomized interventional CCP clinical trial, No./total No. (%)^a^	21/36 (58.3)^b^

Abbreviations: CCP, COVID-19 convalescent plasma; EAP, expanded access program.

^a^ Top-funded NIH sites are those in the top 100 recipients of NIH funding in fiscal year 2020; 36 of the top 100 sites participated in the CCP EAP.

^b^ Percentages for top-funded NIH sites are calculated out of the total number of participating CCP EAP sites in that category.

**Figure.**
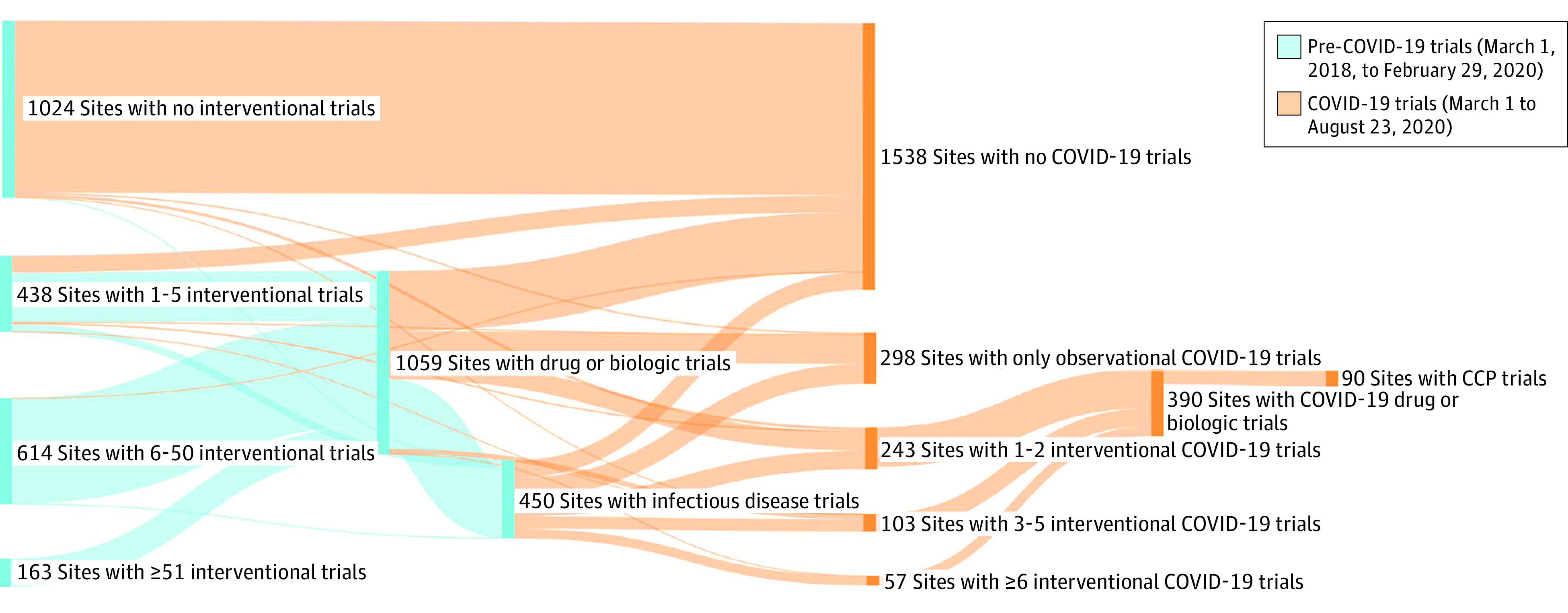
Association Between Trial Types Initiated at COVID-19 Convalescent Plasma (CCP) Early Access Program (EAP) Sites Before and During the COVID-19 Pandemic Blue vertical bars indicate the number of CCP EAP sites that initiated pre–COVID-19 trials of the specified type from March 1, 2018, to February 29, 2020. Orange vertical bars indicate the number of CCP EAP sites that initiated COVID-19 trials of the specified type from March 1 to August 23, 2020. Flow patterns indicate the portion of sites shared across trial types. (Randomized clinical trial design and trial phase are not reflected in this figure.)

## Discussion

The CCP EAP generated important safety information across a diverse group of patients.^3^ However, 2022 treatment guidelines recommend against using CCP for some patients and reflect uncertainty about its use for others.^4,5^

There were several barriers to running CCP trials early in the pandemic, including rolling patient numbers geographically, lack of tests to measure CCP antibodies, and difficulties sourcing CCP with standardized titers.^6^ Expanded access program investigators explained that most participating facilities “would never have been part of a clinical trial.”^6^ Our data support this, given that two-thirds of sites initiated no COVID-19 trials before emergency use authorization of CCP, and nearly half hosted no trials in the 2 years before the pandemic. This suggests that the EAP provided a treatment option many viewed as promising for patients otherwise unable to access it.

The EAP also included sites with substantial research capacity: more than one-third of top 100 NIH-funded institutions and several hundred sites running phase 3 COVID-19 trials. Some of these sites may have chosen the relative ease of the EAP compared with the burden of research—with either CCP or other COVID-19 treatments—including the possibility of patient randomization. However, we do not know how many EAP patients enrolled at these high-capacity sites, and some sites were simultaneously involved in CCP trials. Our analysis is also limited by lack of detailed site information, such as size, type, patient population, and timeline of local COVID-19 surges, as well as methodological challenges matching site names between the EAP and ClinicalTrials.gov. In addition, site involvement in non-CCP trials provides only an estimate of actual CCP trial capacity.

Collaboration between EAP sites with high research capacity to run a large, randomized CCP trial might have rapidly produced stronger answers about whether and for whom CCP is a good therapeutic option. Alternative designs, such as pragmatic cluster crossover trials, could also help address low research capacity. During current and future pandemics, trials should be prioritized and EAPs carefully managed to avoid interference with evidence generation.
